# The Attention Network Test Database: ADHD and Cross-Cultural Applications

**DOI:** 10.3389/fpsyg.2020.00388

**Published:** 2020-03-27

**Authors:** Swasti Arora, Michael A. Lawrence, Raymond M. Klein

**Affiliations:** Department of Psychology and Neuroscience, Dalhousie University, Halifax, NS, Canada

**Keywords:** Attention Network Test, attention, meta-analysis, database, attention deficit/hyperactivity disorder, cross-cultural

## Abstract

Attention is a central component of cognitive and behavioral processes and plays a key role in basic and higher-level functioning. Posner’s model of attention describes three components or networks of attention: the alerting, which involves high intensity states of arousal; the orienting, which involves the selective direction of attention; and the executive control, which involves cognitive functions such as conflict resolution and working memory. The Attention Network Test (ANT) is a computerized testing measure that was developed to measure these three networks of attention. This project describes the ANT, its widely used variants, and the recently developed ANT Database, a repository of data extracted from all studies that have used the ANT as of 2019. To illustrate the potential uses of the database, two meta-analyses conducted using the ANT Database are described. One explores task performance in children with and without attention deficit/hyperactivity disorder (ADHD). The other one explores regional differences between studies conducted in China, Europe, and the United States. We are currently in the process of integrating the database into a publicly available web interface. When that work is complete, researchers, clinicians, and the general public will be able to use the database to explore topics of interest related to attention.

## Introduction

The development and maintenance of attentional capacity is central to cognitive performance. Attentional functioning plays a crucial role in task performance in the daily facets of life, and impairments in attention are present in a multitude of psychiatric disorders such as Alzheimer’s, Parkinson’s, stroke, and schizophrenia (Van Zomeren and Brouwer, 1994; Hyndman and Ashburn, 2003; Minzenberg et al., 2004). In a seminal 1990 article, Posner and Petersen, 1990 describe the components of attention as independent and autonomous from other motor and cognitive components of the brain, such that attention could be directed independently to and concurrently with other operations. The multi-faceted components of attention described in this paper have since been referred to as alerting, orienting, and executive functioning.

**FIGURE 1:**
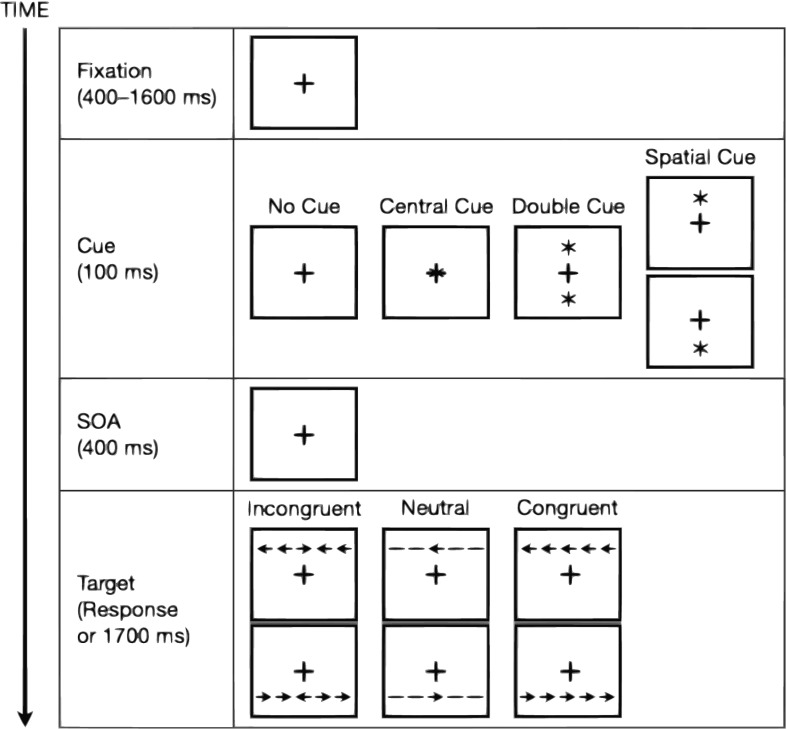
A depiction of the experimental procedure of the original Attention Network Test (ANT) alongside all possible stimuli associated with each event. Redrawn from MacLeod et al. (2010).

The Attention Network Test (ANT) is a tool developed in 2002 by Fan et al. (2002) to assess these three components of attention. This test is a computerized task (Figure 1) that presents a sequence of visual stimuli. The participant is shown cues in the form of one or two asterisks, which can be used to predict an upcoming target presentation and/or be informative of the target’s location. The cue is followed by the presentation of target arrows, which can appear individually or in a sequence of an array of five arrows. Participants are to respond by indicating directionally which way the central arrow is facing, and scores for each network of attention are then calculated by reaction time (RT) subtractions of different stimulus combinations.

One component of attention is the alerting network, which corresponds to the achievement and maintenance of an alert and vigilant state. Activation in the right frontal and parietal hemispheres in the brain occurs when completing tasks that involve the alerting network (Coull et al., 1996; Fan et al., 2005). In the ANT, alerting is measured by the presence versus absence of a visual cue presented before the target. Specifically, it is the subtraction of the mean RT scores for the double cue condition, which alerts participants of the upcoming target, from the mean RTs of the no-cue condition.

Orienting is the ability to selectively attend to a sensory pathway. Overt orienting involves sensory–motor engagement to stimuli, such as the redirection of head and eye movements (Posner, 1980). Covert orienting, which the ANT seeks to explore, involves the internal redirection of processing resources, such as an awareness of stimuli in the peripheral view. Upon presentation of a spatial cue in orienting tasks, activation in the frontoparietal regions of the brain has been shown (Fan et al., 2005). Neuronal activity in the frontal and parietal regions occurs in both overt and covert tasks, however, it has been suggested from studies using single-unit recordings that different neurons within these regions are responsible for mediating overt and covert orienting (Sato and Schall, 2003; Thompson et al., 2005). In the ANT, orienting is measured by the subtraction of the mean RT of the spatially informative cue condition, which both alerts participants of an upcoming target and provides, with 100% validity, the spatial location where the target will appear, from the mean RT of the central cue condition, which only alerts participants of the upcoming target.

Executive control is a term that encompasses many functions, such as problem solving, decision making, self-regulation, and conflict resolution. The ANT targets the latter of these by measuring the participant’s ability to ignore distractor stimuli. This component of attention presents with activation in the anterior cingulate gyrus and dorsolateral prefrontal cortex (Bush et al., 2000; MacPherson et al., 2002; Fan et al., 2005). Network scores for this component of attention are calculated by subtraction of the mean RT of congruent target trials from the mean RT of incongruent target trials.

Following the development of the ANT, a number of variants of the task emerged. Rueda et al. (2004) developed the ANT-C, to assess the networks of attention in child populations. This version of the ANT aimed to be a more engaging and visually stimulating variant that would be more interesting to children. The task was displayed on a neon blue background and used yellow cartoon fish as targets in place of the arrows. The cue preceding the target was displayed for 150 msc, and the fixation period following the presentation of the cue was displayed for 450 msc (both 50 msc longer than in the original ANT). Participants responded with a keyboard button press or on a mouse pad to indicate whether the target was facing left or right. Auditory and visual feedback was presented for correct and incorrect responses. The task also implemented feedback to target responses, in which the participant would hear the sound “Woohoo!” when correctly identifying the orientation of the target or a buzzer sound when making an incorrect response. The fish targets were animated to blow bubbles after a correct response or to have a frown when the response was incorrect. This variant of the ANT has been widely used and is the gold standard of this testing measure in developmental populations.

The Attention Network Test for Interactions (ANT-I) introduced an auditory stimulus (a 2,000 Hz tone presented for 50 ms and 450 ms prior to the presentation of the target) to generate alerting and made the peripheral cue uninformative. The implementation of the auditory cue was designed to allow exploration of the interaction between the alerting and orienting networks (Callejas et al., 2004). The use of a 100% informative peripheral cue in the original ANT was untoward because, being informative, it would elicit endogenous control of attention, but being in the periphery, it would also elicit exogenous control. Endogenous attention is self-directed, whereas exogenous attention is reflexive. Callejas et al. (2004) avoided this conflation of these two modes of control by focusing on the exogenous form of attention that is typically studied by using uninformative peripheral cues. This version of the ANT calculates alerting by a subtraction of the mean RT of the auditory cued trials from the mean RT of trials with no auditory cue. The orienting score is calculated by a subtraction of the mean RT of trials with targets presented at the cued location from the mean RT of trials with targets presented at the uncued location. The executive network, as in the original ANT, is calculated by the subtraction of incongruent trials from congruent trials. This version of the ANT has brought to light the associations between the alerting and orienting networks and provides researchers an alternative version of the ANT task to assist in the independent measurements of these networks (e.g., see Ishigami and Klein, 2010).

A multitude of other variations to the ANT have been developed in subsequent years. The L-ANT or Lateralized ANT was first developed in 2008 by Greene et al., 2008. This task presents targets on a horizontal plane rather than vertical, and it identified that each hemisphere in the brain could support the networks of attention. Although other variants of the ANT have been developed, such as the revised ANT (ANT-R) (Fan et al., 2009) or the vigilance ANT-I (ANTI-V) (Roca et al., 2011), presently, these variants do not figure prominently in the database that will be described next (for a review of the evolution of these ANTs, see De Souza et al., 2020).

### The ANT Database

**FIGURE 2:**
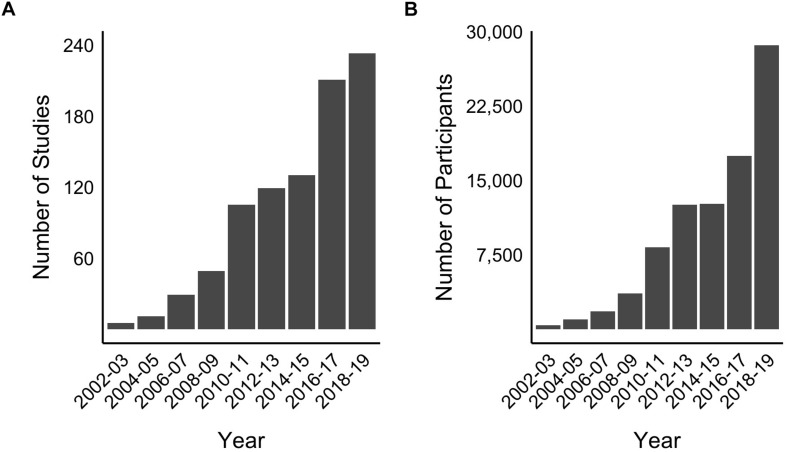
**(A)** Number of studies that used the ANT or a variant of the ANT and **(B)** number of participants tested in these studies.

In our initial exploration (in 2016) of the impact and prevalence of the ANT, over 2,000 articles were identified that had cited the seminal Fan et al. paper. We then sought to explore how many participants had participated in experiments using this testing measure or one of its variants to explore the growing interest in the use of this tool (Figure 2). This preliminary exercise required the review of all articles that had cited the original ANT to identify if they had used the ANT measure and to record the number of participants each study had used. This provided the framework from which the ANT Database was developed. Information pertaining to various metrics was extracted and recorded into an excel worksheet. Data corresponding to the network scores for each of the three attentional networks, overall mean RT, and corresponding mean standard deviation/error, and error rate were also extracted.

Notably, at its current iteration, the database has been collated from over 3,000 studies that have cited the seminal ANT paper. The purpose of this paper is to describe this database and to illustrate its usefulness with some examples. It is our plan to make the database available to researchers, clinicians, and the general public via a web-based interface, the development of which is underway. This resource has allowed us to conduct two meta-analyses to date: explorations of attentional performance in children with and without attention deficit/hyperactivity disorder (ADHD), and regional differences in attentional processing between participants residing in China, Europe, and the United States. As previously described in Figure 2, the number of studies using the ANT is growing at an accelerated rate and presents the potential for extensive and robust meta-analytic research facilitated by the database.

## Materials and Methods

The ANT Database was generated using the PubMed, Google Scholar, and Web of Science search engines with a comprehensive search of articles that had cited the original Fan et al. (2002) paper, “Testing the Efficiency and Independence of Attentional Networks.” This search yielded 3,179 articles published in or prior to 2019. Inclusion criteria required the methods section of each article that mentioned the “attention network test” or “attention network task” in the title or abstract to be reviewed manually to see if an ANT testing measure was used with participants, rather than merely referenced or described. If the description of an experiment in the abstract suggested that an attention testing measure was used, the methods section of the article was assessed for eligibility. After the removal of duplicates, we excluded review papers, commentaries, and studies that described but did not use the ANT. This method of selection yielded 889 individual studies that collected data using the ANT or one of its variants. This method of systematic review is similar to the guidelines described in the Preferred Reporting Items for Systematic Reviews and Meta-Analysis (PRISMA) Group Statement but did not organize and classify studies that were excluded due to ineligibility (Moher et al., 2009).

Data extraction was completed manually, and information pertaining to the purpose of the study, participant demographics, condition or clinical subset, number of participants in each condition, geographical region, and version of the ANT were entered into a spreadsheet. Error rates, mean RTs, and corresponding standard deviations/errors were extracted when provided. RTs and standard deviations/errors were extracted from the results section, supplementary tables, and, if not provided in the text, data figures using the WebPlotDigitizer, a web-based extraction tool (Rohatgi, 2011). To date, we have extracted data from 382 of 868 studies in the database. The web interface for the ANT Database is hosted at http://attentionnetwork.ca/.

## Application 1: Developmental Adhd

A meta-analysis was performed on studies having conducted research with a clinical population of children with a diagnosis of ADHD and matched neurotypical healthy controls. Children with ADHD present with symptoms of consistent challenges with sustained attention, hyperactive behavioral tendencies, and challenges in organization and short-term memory consolidation.

Despite the concerns of increased rates of ADHD that have been brought forth, the prevalence of children who meet diagnostic criteria has been shown to be relatively consistent over the past three decades across major geographical regions (Polanczyk et al., 2014). However, research in the United States has shown ADHD diagnoses to have a positive relationship with lower socioeconomic status and gender, with boys having higher rates of diagnosis (Froehlich et al., 2007). Some argue that this is indicative of how ADHD and behavioral symptoms are presented across genders, with boys presenting with more frequent externalizing behaviors in contrast to common internalizing behaviors seen in girls, such as inattentiveness (Quinn and Madhoo, 2014).

Impairments in executive functioning have been proposed to be associated with ADHD over the past three decades (Denckla, 1989). Children with ADHD present with high rates of executive functioning deficits in comparison to neurotypicals, which have been found to increase the likelihood of experiencing academic difficulties (Biederman et al., 2004). These findings have been consistently replicated in children and adults through various experimental explorations (Barkley and Murphy, 2010; Holmes et al., 2010; Schoemaker et al., 2012; Silverstein et al., 2018) and are considered an area of focus for behavioral modification practices (Diamond and Lee, 2011). Prior to the development of the ANT, Berger and Posner (2000) specifically asserted that people with ADHD should not show deficits in orienting but should have deficits in alerting and executive control. If we regard this as a prediction, this meta-analysis can be viewed as a test.

### Methods: ADHD

This meta-analysis consisted of response time data (accuracy data were reported by too few studies) from studies that had both a control and an ADHD group, with participants in both groups ranging from 6 to 13 years of age. One study, Johnson et al. (2008), did not report age ranges, however, based on their mean age and SD, 95% of participants fell between 8 and 18 years of age. Studies used the ANT, modified ANT, L-ANT, or ANT-C. This resulted in 491 participants with ADHD and 402 controls, as described in Table 1. One study (Konrad et al., 2010) in this meta-analysis did not report their standard deviations or standard errors. The mean standard deviation of the remaining eight control groups and 11 ADHD groups was calculated and substituted in place.

**TABLE 1:** Participants demographics in the child attention deficit/hyperactivity disorder (ADHD) meta-analysis.*

	ADHD		Control	
		
Study	Participants	Mean age	Participants	Mean age
Adólfsdóttir et al. (2008)	45	10.00	57	10.00
Booth et al. (2007)	26 (ADHD-I)	9.501.83	24	9.501.58
	16 (ADHD-C)	9.501.83	–	–
Hansen et al. (2014)	38	9.801.50	35	10.702.40
Heinrich et al. (2014)	15 (ADHD-C)	9.811	19	10.161.05
	9 (ADHD-I)	9.380.99	–	–
Johnson et al. (2008)	73	12.702.30	73	13.101.90
Konrad et al. (2010)	181	11.50	121	10.20
Konrad et al. (2006)	16	10.201.90	16	10.101.30
Kooistra et al. (2011)	16 (ADHD-I)	9.700.85	38	9.131.12
	31 (ADHD-C)	9.081.93	–	–
Kratz et al. (2011)	25 (ADHD-I & ADHD-C)	9.701.00,	19	10.201.00
		9.601.10		

*^∗^ADHD-C and ADHD-I denote individuals in the “Combined” and “Inattentive” subsets.*

### Results: ADHD

**FIGURE 3:**
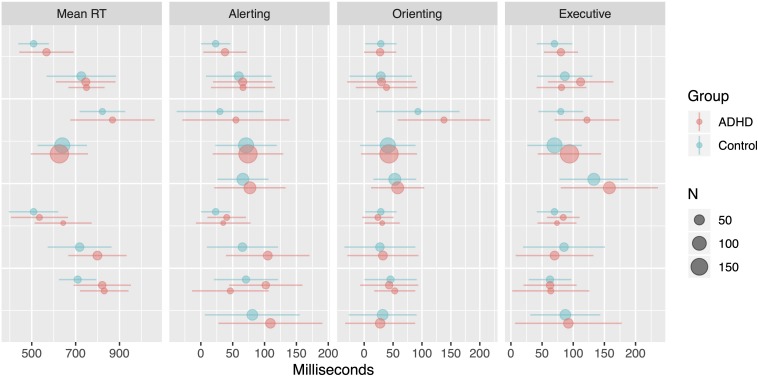
Forest plot of data used for meta-analysis. Dots reflect means sized by N, and lines reflect ± 1SD. Data from the same study are grouped together vertically.

Mean age weighted by the number of participants in a study was calculated for the ADHD group (10.8 ± 1.58 years) and the control group (10.6 ± 1.53 years). Forest plots for each study in this meta-analysis are presented in Figure 3. Data points represent the mean network score or overall mean RT for the ADHD or control group in each study. Quantitative inference was achieved through a Bayesian hierarchical (a.k.a. “multi-level”) model, coded and sampled using Stan (Carpenter et al., 2017). Since each study observed at least one group of control and ADHD children (three studies observed multiple ADHD groups differing by diagnosis subtype; these were treated as repeated observations in the model), we are able to model the across-groups average (a.k.a. “intercept”) for each measure (mean RT, alerting, orienting, and executive) as well as the between-groups difference on each measure as normally distributed across studies (a.k.a. a “partially pooled” model, a.k.a. a “random effects” model), using the observed standard errors for each study to specify its respective measurement noise. Weakly informed priors were employed, and posterior samples were obtained across six independent chains, each consisting of 10,000 warmup and 10,000 post-warmup iterations. All models passed all diagnostic checks provided by the rstan package (Stan Development Team, 2019) for R.

**FIGURE 4:**
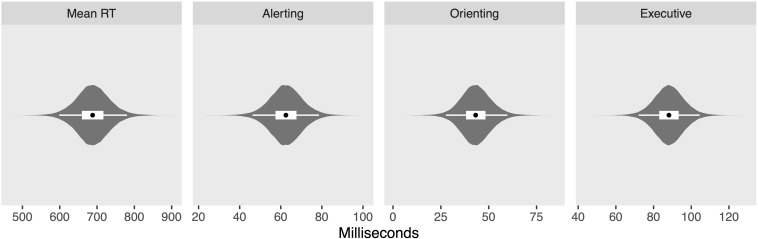
Violin plots of posterior distributions for intercepts. Black dots reflect posterior median, thick white band reflects 50% credibility interval (CrI), and thin white band reflects 95% CrI.

**FIGURE 5:**
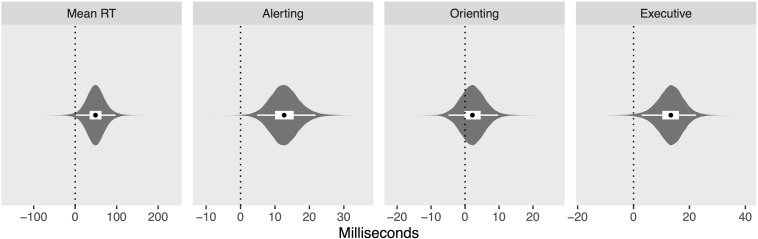
Violin plots of posterior distributions for between-group differences, attention deficit/hyperactivity disorder (ADHD) minus control. Black dots reflect posterior median, thick white band reflects 50% CrI, and thin white band reflects 95% CrI.

Figure 4 shows the posterior distribution of the intercepts for overall mean RT (posterior median: 688 ms; 95% credibility interval (CrI): [598, 780]), alerting (62 ms; [46, 79]), orienting (43 ms; [28, 60]), and executive (88 ms; [72, 104]) networks, while Figure 5 shows the posterior distribution for the between-groups differences (ADHD minus controls) for overall mean RT (49 ms; [2, 97]), alerting (13 ms; [5, 22]), orienting (2 ms; [−5, 10]), and executive (13 ms; [3, 22]) networks. From these results, we may conclude that the difference between groups is credibly non-zero for all measures except the orienting network.

**FIGURE 6:**
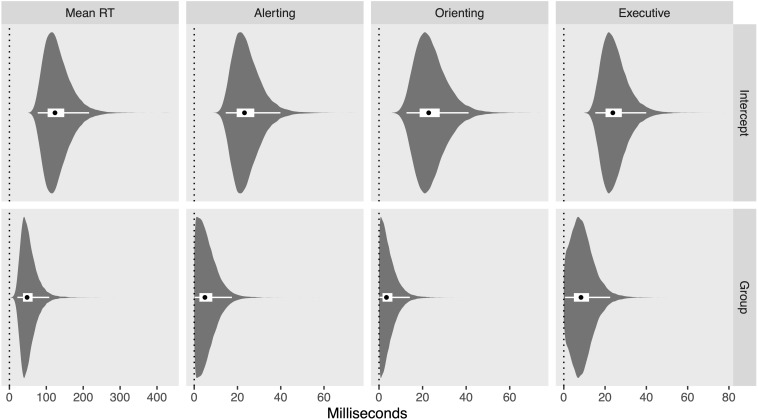
Violin plots of the posterior distribution for heterogeneity parameters. Black dots reflect posterior median, thick white band reflects 50% CrI, and thin white band reflects 95% CrI.

The hierarchical model also permits quantification of heterogeneity between studies for each measure, reflecting how much the studies differ from one to another over and above mere measurement error. Such differences would be attributable to unmodeled differences between the studies’ respective methods (e.g., instruction emphasis on speed vs. accuracy, time of day, etc.) or sample populations (e.g., age range, socioeconomic status, etc.). Accounting for this heterogeneity is one of the primary benefits of the hierarchical model employed here, and this modeling choice is validated to the degree that the posterior distribution on the heterogeneity parameter is shifted away from zero. Figure 6 depicts posterior distributions on heterogeneity for both the intercept and the difference between groups for each measure, revealing credibly non-zero heterogeneity for all intercepts as well as the difference between groups on mean RT, while zero heterogeneity remains relatively credible for the difference between groups on alerting, orienting, and executive network scores.

### Discussion: ADHD

The findings from our ADHD meta-analyses are consistent with Berger and Posner (2000) analysis and much of the literature on this clinical subset. Impairments in the alerting and executive networks appear to be present in children with developmental ADHD compared to neurotypical controls (Cao et al., 2008; Lambek et al., 2011). These findings demonstrate that these differences in attentional processing are consistent across the regions in which these studies were conducted, such as Canada, the United States, Germany, and Norway.

Using the ANT-I (not included in this meta-analysis due to the difference in control of orienting from the standard ANT), Mullane et al. (2011) found similar group differences in the alerting and executive networks. Moreover, using purely exogenous cues (as compared to the hybrid cues used in the ANT), they also found no group difference in the orienting network. Converging evidence for an absence of an orienting deficit, under both exogenous and endogenous control, in individuals with ADHD, can be found in a 2003 review (Huang-Pollock and Nigg, 2003) and in a recent paper using perceptual measures with adult participants (Roberts et al., 2018). As previously described, one study in this meta-analysis explored familial differences in attention with children with ADHD and their parents and siblings (Konrad et al., 2010). Given the proposed familial heritability of ADHD symptoms, we examined network scores excluding this study and found no significant changes in network scores from those reported in our results.

In structural MRI studies, brain regions associated with the alerting and executive networks have been shown to be different in individuals with ADHD (De La Fuente et al., 2013). The orbitofrontal and superior frontal cortices, which have been shown to have activation in tasks involving alerting, present with reduced activation and reduced cortical density in developmental ADHD (Shaw et al., 2006; Cubillo et al., 2010), while the dorsolateral prefrontal cortex (DLPFC), which is associated with executive control, has fractional anisotropy abnormalities and a reduced relative size in comparison to control participants (Makris et al., 2008). These reductions in activation and relative size of brain structures may play a role in the behavioral presentation of the attentional processing differences present in ADHD.

## Application 2: Cross-Cultural

Differences in linguistics and parental interactions have been shown to exist across cultures. Posner and Rothbart (1994), for example, noted that caregivers of infants in Western cultures are more likely to use redirecting behaviors to console children. Such early interactions may play a role in shaping and priming the development of human attention. Caregivers’ emphasis on labeling objects in Western cultures compared to the emphasis on social interactions in Eastern cultures has been proposed to play a role in cultural variation of perception (Nisbett and Miyamoto, 2005). These variations are also present in linguistic differences in Mandarin- and English-speaking toddlers, with the former using more verbs and the latter more nouns (Tardif et al., 1999). These differences in object focus might influence the development of the networks of attention.

The orienting network develops as a function of socialization within culture and community. Executive functioning, which involves the voluntary facilitation of attention, has apparent connections to an individual’s cultural environments (Posner and Rothbart, 2017). Given these previously proposed social distinctions between the components of attention, regional differences might be expected to be present in these networks. On the other hand, Posner and Rothbart (2017) have suggested that the alerting network may be a more purely biological function. In that case, cross-cultural differences in alertness might not be observed.

### Methods: Cross-Cultural

This meta-analysis explored cross-cultural differences in the three networks of attention. Regions in which the studies were conducted were identified through the methods section of each paper. Studies that had used the ANT were then grouped together by region and matched by age and number of participants. This resulted in 27 studies in total and nine from each region (China, Europe, and the United States). Each region had a combined number of 330 ± 5 participants. A detailed breakdown of the demographics and sources for this meta-analysis is presented in Table 2. Only the control condition of an experiment was included in this meta-analysis, and all studies reported network scores and standard deviations of these scores. Overall, mean RT was calculated from the seven studies that reported overall mean RT in China, eight studies in Europe, and four studies (of which one did not report SD for mean RT) from the United States.

**TABLE 2:** Participant demographics in the cross-cultural meta-analysis.

	Participants	Age
**China**		
Fang et al. (2014)	18	40.3710.70
Lv et al. (2010)	45	64.785.15
Liu et al. (2013)	30	23.501.20
Liu et al. (2014)	26	40.5511.96
Tian et al. (2016)	30	34.2012.20
Wang et al. (2005)	53	29.4212.63
Yuan et al. (2014)	42	30.888.61
Zhang et al. (2015)	20	19.890.88
Zhou et al. (2011)	30	27.805.63
Zhou et al. (2011)	30	51.205.82
**Total:**	324	
**Europe**		
Breton et al. (2011)	53	43.7712.18
Hahn et al. (2011)	55	33.609.20
Jo et al. (2016)	20	40.808.60
Jongen et al. (2015)	23	26.903.40
Lundervold et al. (2011)	56	29.207.10
Neuhaus et al. (2011)	18	27.392.70
Neuhaus et al. (2011)	18	40.947.30
Neuhaus et al. (2010)	44	30.397.10
Schötz et al. (2016)	20	39.708.41
Zajenkowski et al. (2015)	28	27.404.50
**Total:**	335	
**United States**		
Fergus and Carleton (2016)	86	18.901.10
Gooding et al. (2006)	24	39.7914.30
Hubbard et al. (2015)	14	31.0010.91
Kaufman et al. (2016)	19	22.904.00
Myers et al. (2013)	30	29.808.50
Nestor et al. (2007)	30	41.278.59
Roth et al. (2015)	40	46.208.70
Tully et al. (2012)	23	28.7613.81
Woicik et al. (2009)	63	38.706.50
**Total:**	329	

### Results: Cross-Cultural

**FIGURE 7:**
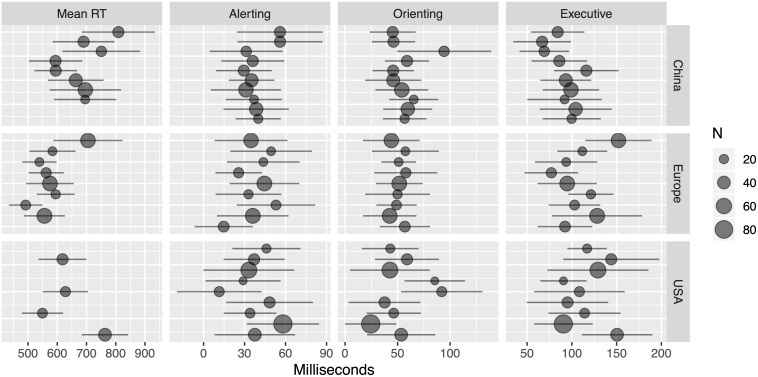
Forest plot of data used for meta-analysis. Dots reflect means sized by N, and lines reflect ± 1SD.

Mean age, weighted by the number of participants in a study for China (37.2 ± 8.57), Europe (33.9 ± 7.55), and the United States (32 ± 9.4), was similar across all three regions. Forest plots for each study in the cross-cultural meta-analysis are presented in Figure 7.

As in the previous application, quantitative inference was achieved through a Bayesian hierarchical model, coded and sampled using Stan (Carpenter et al., 2017). Each combination of region (China, Europe, United States) and measure (mean RT, alerting, orienting, executive) was modeled independently as forming a normal (i.e., partially pooled) distribution across studies, using the observed standard errors for each study to specify its respective measurement noise. Weakly informed priors were employed, and posterior samples were obtained across six independent chains, each consisting of 10,000 warmup and 10,000 post-warmup iterations. All models passed all diagnostic checks provided by the rstan package (Stan Development Team, 2019) for R.

**FIGURE 8:**
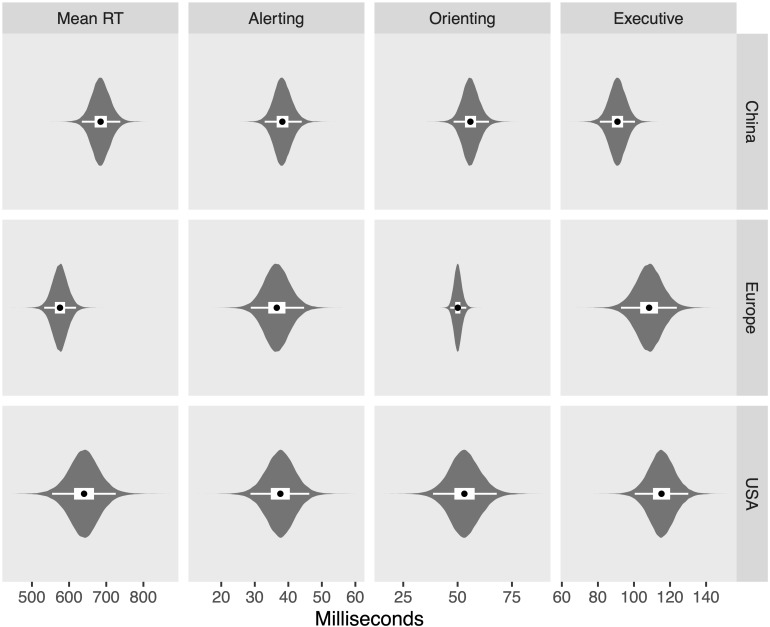
Violin plots of posterior distributions. Black dots reflect posterior median, thick white band reflects 50% CrI, and thin white band reflects 95% CrI.

**FIGURE 9:**
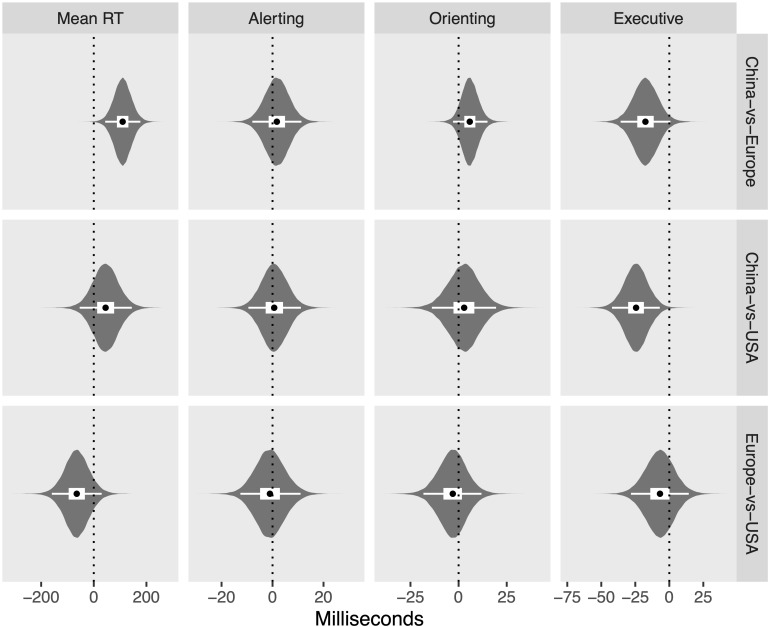
Violin plots of posterior distributions for between-group differences. Black dots reflect posterior median, thick white band reflects 50% CrI, and thin white band reflects 95% CrI.

**FIGURE 10:**
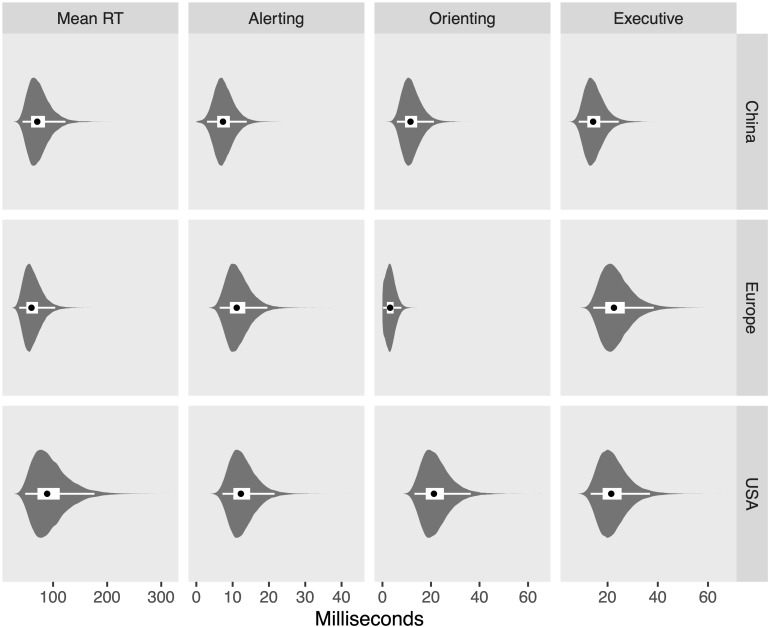
Violin plots of posterior distributions for heterogeneity between studies. Black dots reflect posterior median, thick white band reflects 50% CrI, and thin white band reflects 95% CrI.

The posterior distributions for each measure and region are shown in Figure 8, while Figure 9 shows the differences between regions on each measure, in which zero remains relatively credible for all differences, with the exception of the difference between China and Europe in mean RT (110 ms; [43, 177]) and the difference between China and the United States in executive control (−24 ms, [−42, −7]). Finally, Figure 10 shows the posterior distributions of the heterogeneity between studies for each region and measure, showing credibly non-zero heterogeneity for all combinations, with the exception of orienting scores measured in Europe.

### Discussion: Cross-Cultural

The differences in the cross-cultural exploration have intriguing epistemological implications and suggest regional variations in attentional processing. Consistent with the suggestion (Posner and Rothbart, 2017) that alerting is primarily biologically determined the meta-analysis didn’t reveal any regional/cultural differences in the alerting network scores. In fact, zero remained a relatively credible value for alerting and orienting comparisons in the three regions. There were, however, regional/cultural differences in the executive networks, with participants in China showing smaller executive scores (reflecting more efficient filtering) than those from the United States and trends suggesting similar differences between participants from Europe.

Analytical vs. holistic thinking style differences may account for the variations in the executive networks found in this meta-analysis. Chinese participants have been shown to have a higher sensitivity to discrepant stimuli (as demonstrated by increased P3 amplitudes in a three-stimulus oddball design), whereas European participants have increased P3 responses to designated target events (Lewis et al., 2008). This difference in visual focus may play a role in the smaller executive network scores of Chinese participants by permitting faster consolidation and then filtering of peripheral flankers. The aforementioned differences in child-rearing styles across cultures may also influence the development of attentional networks (Gutchess et al., 2006; Boduroglu et al., 2009). Building on their previous observation (Posner and Rothbart, 1994) of redirective soothing behaviors in Western cultures, Posner and Rothbart proposed that Western participants might attend faster and more frequently to focal objects (Posner and Rothbart, 2017). In this application, there would have been significant methodological differences within groups between studies, given that each study could only provide data for a single group. To further explore this topic, a larger sample size could be generated following developments in the database.

## General Discussion

As illustrated by the two applications presented here, this database has a broad reach and can be utilized for many meta-analytic explorations. It is our intention to further develop and maintain this database and make it accessible for public use through a web interface. This resource will present users with public health information pertaining to attention and how it is affected in a multitude of clinical conditions and by various experimental manipulations (including the effects of pharmaceuticals, sleep deprivation, exercise, etc.). In the impetus for open science, the ANT Database will allow users to conduct meta-analyses on topics of their interest by centralizing the data extracted.

As the meta-analytic explorations conducted through the database have been through the secondary use of data, various limitations are present. Participants in the regional explorations had been selected from the control condition of a multitude of studies, for which differences in task implementation cannot be controlled. Variables such as socioeconomic status and participant age may also play a role, while general age across the three regions was relatively consistent. Within the ADHD exploration, studies consisted of experiments that had participants from both conditions, who were roughly the same age (10.6 and 10.8), and who would have experienced identical tasks. However, standard deviation or error was not reported in all studies. While the stand-in standard deviation calculated from the remaining studies served as an adequate estimate, there is room for error in this approximation.

The applications and utility of the database are extensive, and it can be a useful tool for conducting further meta-analyses and providing a resource to attention researchers, clinicians, and knowledge users. In older adults, attention performance has been shown to deteriorate with age, and impairments in visual attention are predictive of other cognitive and neurological decline (Owsley and McGwin, 2004). While promising interventions such as exercise and meditation have been shown to be effective in attenuating the effects of cognitive decline and improve self-regulation in young and older adults (Tang et al., 2014), future applications with the database could explore these developmental differences in attention. Other topics frequently discussed in the database are clinical impairments in individuals with depression and anxiety, which could be further explored across different parameters.

The next step in this project will be to further develop the web interface of the database. Our initial focus will be to continue the data extraction of the remaining studies and bring the database up to date; however, future directions will be to create a tool for public health and global applications of attention. This interface will provide clinicians, patients, and interested parties a centralized resource and detail recent findings on topics of interest identified by public users that will be highlighted on the web interface. This resource will minimize the expenditure of researchers and clinicians to provide literature and resources to patients with attentional impairments, provide information to knowledge users in an easy-to-interpret way, and foster interdisciplinary and community collaboration.

## Data Availability Statement

The data sets and analysis scripts for this study are available at (https://osf.io/edfqn/). The web interface for the ANT Database described in this manuscript is hosted at http://attentionnetwork.ca (presently under development).

## Ethics Statement

Ethical review and approval was not required for the study on human participants in accordance with the local legislation and institutional requirements. Written informed consent for participation was not required for this study in accordance with the national legislation and the institutional requirements.

## Author Contributions

RK and SA contributed to the conception and design of this project. This research was conducted by SA under the direct supervision, guidance, and support of RK. ML performed statistical analysis and modeling of the data. All authors contributed to manuscript revision, and read and approved the submitted version.

## Conflict of Interest

The authors declare that the research was conducted in the absence of any commercial or financial relationships that could be construed as a potential conflict of interest.
